# Multimodal Imaging with NanoGd Reveals Spatiotemporal Features of Neuroinflammation after Experimental Stroke

**DOI:** 10.1002/advs.202101433

**Published:** 2021-07-01

**Authors:** Violaine Hubert, Ines Hristovska, Szilvia Karpati, Sarah Benkeder, Arindam Dey, Chloé Dumot, Camille Amaz, Naura Chounlamountri, Chantal Watrin, Jean‐Christophe Comte, Fabien Chauveau, Emmanuel Brun, Patrice Marche, Fréderic Lerouge, Stéphane Parola, Yves Berthezène, Thomas Vorup‐Jensen, Olivier Pascual, Marlène Wiart

**Affiliations:** ^1^ Univ‐Lyon IRIS Team CarMeN Laboratory Inserm U1060 INRA U1397 INSA Lyon Université Claude Bernard Lyon 1 Groupement Hospitalier Est 59 bd. Pinel Bron 69500 France; ^2^ SYNATAC Team Institut NeuroMyoGène Université Claude Bernard Lyon 1 CNRS UMR 5310, INSERM U1217 Faculté de Médecine et de Pharmacie 8 avenue Rockefeller Lyon 69008 France; ^3^ Université de Lyon École Normale Supérieure de Lyon CNRS UMR 5182 Université Claude Bernard Lyon 1 Laboratoire de Chimie Lyon F69342 France; ^4^ Institut pour l'Avancée des Biosciences Centre de Recherche UGA / Inserm U 1209 / CNRS UMR 5309 Site Santé ‐ Allée des Alpes La Tronche 38700 France; ^5^ Clinical Investigation Center Hospices Civils de Lyon Louis Pradel Hospital 28 avenue Doyen Lépine Bron 69500 France; ^6^ FORGETTING Team Lyon Neuroscience Research Center (CRNL) CNRS UMR5292 INSERM U1028 Université Claude Bernard Lyon 1 Centre Hospitalier Le Vinatier ‐ Bâtiment 462 ‐ Neurocampus Michel Jouvet 95 boulevard Pinel Bron 69675 France; ^7^ Université de Lyon Lyon Neuroscience Research Center (CRNL) CNRS UMR5292 INSERM U1028 Université Claude Bernard Lyon 1 Groupement Hospitalier Est ‐ CERMEP 59 bd Pinel Bron Cedex 69677 France; ^8^ Synchrotron Radiation for Biomedical Research (STROBE) UA7 INSERM Université Grenoble Alpes Medical Beamline at the European Synchrotron Radiation Facility 71 Avenue des Martyrs Grenoble Cedex 9 38043 France; ^9^ Univ‐Lyon Creatis Laboratory CNRS UMR5220 Inserm U1044 INSA Lyon Villeurbanne Cedex 69621 France; ^10^ Department of Biomedicine Biophysical Immunology Laboratory Aarhus University Aarhus C DK‐8000 Denmark

**Keywords:** intravital two‐photon microscopy, magnetic resonance imaging, microglia/macrophage, multimodal nanoprobe, neuroinflammation, stroke

## Abstract

The purpose of this study is to propose and validate a preclinical in vivo magnetic resonance imaging (MRI) tool to monitor neuroinflammation following ischemic stroke, based on injection of a novel multimodal nanoprobe, NanoGd, specifically designed for internalization by phagocytic cells. First, it is verified that NanoGd is efficiently internalized by microglia in vitro. In vivo MRI coupled with intravenous injection of NanoGd in a permanent middle cerebral artery occlusion mouse model results in hypointense signals in the ischemic lesion. In these mice, longitudinal two‐photon intravital microscopy shows NanoGd internalization by activated CX3CR1‐GFP/+ cells. Ex vivo analysis, including phase contrast imaging with synchrotron X‐ray, histochemistry, and transmission electron microscopy corroborate NanoGd accumulation within the ischemic lesion and uptake by immune phagocytic cells. Taken together, these results confirm the potential of NanoGd‐enhanced MRI as an imaging biomarker of neuroinflammation at the subacute stage of ischemic stroke. As far as it is known, this work is the first to decipher the working mechanism of MR signals induced by a nanoparticle passively targeted at phagocytic cells by performing intravital microscopy back‐to‐back with MRI. Furthermore, using a gadolinium‐based rather than an iron‐based contrast agent raises future perspectives for the development of molecular imaging with emerging computed tomography technologies.

## Graphical abstract

Neuroinflammation at the subacute stage of stroke is challenging to monitor in vivo. Here, a novel gadolinium‐based fluorophore‐grafted nanoprobe named NanoGd, specifically designed for internalization by phagocytic cells, is worked with. An in vivo magnetic resonance imaging (MRI) tool is proposed to monitor neuroinflammation following ischemic stroke in mice, which is validated using two‐photon intravital imaging back‐to‐back with MRI for the first time.

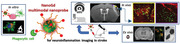

## 1 Introduction

Ischemic stroke is a devastating neurological condition and the second cause of death in Western countries. Neuroinflammation is a major component of stroke pathophysiology, often associated with impaired outcome.^[^ ^1^ ^]^ It is well established that phagocytic cells, including tissue‐resident microglia and blood‐borne recruited macrophages, are the main cellular mediators of inflammation initiation and continuation following stroke. By secreting toxic molecules, they may contribute to tissue damage early in the acute phase of stroke and are thus potential therapeutic targets.^[^ ^2^, ^3^ ^]^

The gold‐standard for imaging immune cells in vivo in preclinical settings is intravital two‐photon microscopy coupled with the use of transgenic mouse lines and/or fluorescent dyes.^[^ ^4^ ^]^ Two‐photon microscopy has been used to investigate the dynamics of brain immune cells and their interaction with the neural environment in several neurological diseases,^[^ ^5^ ^]^ enabling exciting new discoveries in mouse models of focal blood–brain barrier (BBB) disruption,^[^ ^6^ ^]^ traumatic brain injury,^[^ ^7^ ^]^ Alzheimer's disease,^[^ ^8^ ^]^ and stroke.^[^ ^9^, ^10^ ^]^ At present, absorption of light by the skull prevents clinical translation of this method. In addition, only part of the cortex can be imaged due to the low depth of penetration of light. Therefore, a complementary 3D imaging technique is needed to provide information about macrophage activation within the whole brain. Positron emission tomography (PET) associated to radiotracers targeting the translocator protein TSPO, a biomarker of microglia, is the gold‐standard for inflammation imaging in clinical settings. In patients with ischemic stroke, PET imaging reveals inflammatory phenomena both at the lesion site and remotely,^[^ ^11^, ^12^, ^13^ ^]^ but only in chronic stages (>72 h).^[^ ^14^ ^]^ Therefore, it cannot be used to study the early inflammatory response following stroke. In recent decades, contrast agents based on (ultrasmall) superparamagnetic particles of iron oxides ((U)SPIOs) have been developed as magnetic resonance imaging (MRI) biomarkers of neuroinflammation.^[^ ^15^ ^]^ When injected intravenously, (U)SPIOs are internalized by phagocytic cells, which become magnetic and can be detected with MRI. Thus, MRI coupled to intravenous injection of (U)SPIOs is a noninvasive tool for imaging immune cell trafficking across the inflamed central nervous system (CNS).^[^ ^16^, ^17^ ^]^ (U)SPIO‐enhanced MRI has been used to track phagocytic cells, mostly macrophages, in rodent models in the acute and subacute phases of permanent ischemic stroke.^[^ ^18^, ^19^, ^20^, ^21^ ^]^

The present study investigated the potential of a novel gadolinium (Gd)‐based MR contrast agent, NanoGd, for preclinical multimodal in vivo imaging of neuroinflammation after focal cerebral ischemia. NanoGd is a hybrid nanoparticle specifically designed to be internalized by phagocytic cells in vivo. Synthesis and characterization, including multimodal imaging properties, biodistribution and pharmacokinetics, have been reported in detail elsewhere.^[^ ^22^ ^]^ This latter study also showed that NanoGd was efficiently internalized by primary bone‐marrow derived macrophages (BMDMs) without inducing inflammation or cell mortality.^[^ ^22^ ^]^ In the current study, the aim was twofold: 1) to evaluate whether NanoGd‐enhanced MRI would be suited to monitor in vivo phagocytic cells following NanoGd internalization, and 2) to improve the understanding of NanoGd‐related MRI signals using two‐photon intravital imaging back‐to‐back with MRI. Following assessment of NanoGd internalization by microglia in vitro, we assessed the potential of NanoGd as an in vivo biomarker of neuroinflammation in transgenic CX3CR1eGFP/+ mice subjected to permanent ischemic stroke. 3D high‐resolution gadolinium brain mapping, histological staining, and transmission electron microscopy (TEM) were performed postmortem to corroborate the in vivo results.

## 2 Results

### 2.1 NanoGd Is Internalized by Microglia In Vitro without Overt Inflammatory Effects

**Figure 1:**
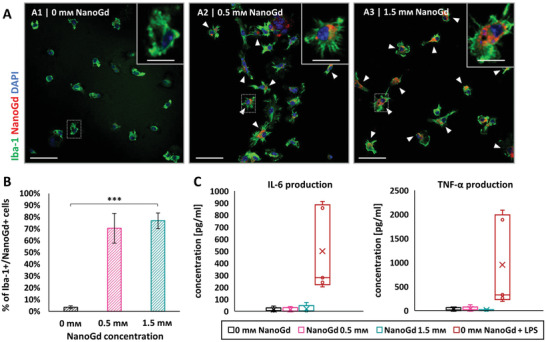
NanoGd is internalized by microglial cells in vitro without increasing proinflammatory cytokine production. A) Confocal images of Iba‐1‐stained microglial cells incubated A1) without or A2,A3) with NanoGd (0.5 mmol L^–1^; 1.5 mmol L^–1^). White arrowheads indicate Iba1+ cells that colocalized with NanoGd (scale bars: 50 µm for overview images; 10 µm for magnified insets). B) Percentage of cultured Iba1+ cells that internalized NanoGd. Data are displayed as mean ± SD. Independent experiments, *n* = 2; replicates per condition, *n* = 3 for each experiment. Significant differences between experimental conditions were calculated on one‐way ANOVA and are indicated by *** for *p* < 0.001. C) Quantification of interleukin‐6 (IL‐6) and tumor‐necrosis factor alpha (TNF‐*α*) production by microglial cells exposed to different concentrations of NanoGd. Cells exposed to LPS but not to NanoGd (condition “0 × 10^−3^ m NanoGd + LPS”) were used as a positive control for production of proinflammatory cytokines. Independent experiments, *n* = 2; replicates per condition, *n* = 3 for experiment 1 and *n* = 2 for experiment 2. On boxplots, the “X” represents the mean and the circles show the interior or outlier points.

To obtain further evidence of NanoGd internalization by phagocytic cells in brain ischemia, we first investigated internalization in primary microglia culture in vitro. NanoGd red fluorescent signal was detected in microglial cultures incubated with either 0.5 × 10^−3^ m(**Figure** **1**A2) or 1.5 × 10^−3^ m of NanoGd (Figure 1A3), but not in control microglial culture without NanoGd (Figure 1A1). Confocal images showed internalization of NanoGd by Iba‐1‐stained cells following NanoGd incubation (Figure 1A2,A3, white arrowheads). Higher magnification revealed cytoplasmic location (Figure 1A2,A3, insets). Quantitative analyses confirmed these results, showing that more than two‐thirds of Iba1+ cells internalized NanoGd following incubation (Figure 1B). We also assessed the production of proinflammatory cytokines interleukine‐6 (IL‐6) and tumor‐necrosis factor‐*α* (TNF‐*α*) by microglial cultures exposed to NanoGd. Results showed that NanoGd exposure did not impact production of IL‐6 and TNF‐*α* compared to nonexposed control cells (Figure 1C). By contrast, microglial cells exposed to liposaccharide (LPS), used as a positive control for proinflammatory cytokine production, produced higher concentrations of IL‐6 and TNF‐*α* (Figure 1C).

### 2.2 NanoGd Accumulation in the Lesion Is Detected In Vivo with MRI

**Figure 2:**
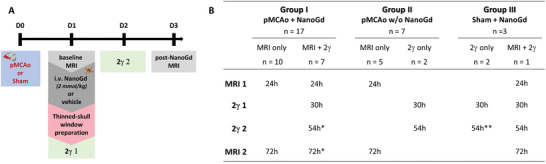
Experimental timeline and study design. A) Experimental timeline designs for NanoGd in vivo multimodal imaging. 2*γ* 1: intravital two‐photon microscopy, imaging session 1; 2*γ* 2: intravital two‐photon microscopy, imaging session 2. B) Summary of imaging exams undergone by each mouse, in 3 groups. MRI 1 represents the baseline pre‐NanoGd session, and MRI 2 the post‐NanoGd MRI session. Time intervals in hours (24, 30, 54, or 72 h) correspond to the exact time of imaging post‐pMCAo. Asterisks correspond to changes in animal number of group I and group III: ^*^ *n* = 6 and ^**^ *n* = 1, both due to the death of one animal during the first two‐photon microscopy session. Of note, statistical analyses have been performed for experimental groups with a sample size superior to *n* = 2. i.v.: intravenous; w/o: without.

To evaluate the potential of NanoGd as an imaging biomarker of neuroinflammation following stroke, we established a longitudinal in vivo imaging protocol. **Figure** **2**A shows the experimental design of the in vivo studies and Figure 2B summarizes the number of animals used in each group and their imaging examinations.

**Figure 3:**
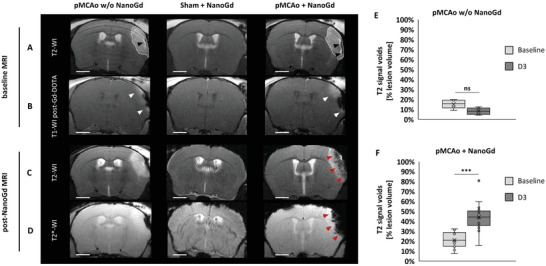
In vivo multiparametric MRI allows the characterization of NanoGd distribution in the ischemic lesion. A,B) Pre‐ and C,D) post‐NanoGd MRI for three representative mice, one pMCAo and one sham‐operated mouse, both injected with NanoGd, and one noninjected pMCAo mouse. For each MRI sequence, only one transversal slice is shown (scale bars: 1 mm). A) T2‐weighted images (T2‐WI) show an ischemic lesion in pMCAo mice (dotted white lines), but not in the sham mouse. Black arrowheads indicate hypointense artefacts associated with pMCAo surgery. B) BBB disruption was assessed on T1‐weighted images (T1‐WI), pre‐ and postinjection of a small gadolinium chelate. T1 enhancement in pMCAo mouse brain was indicative of BBB disruption (white arrowheads). 48 h following NanoGd injection, NanoGd presence at the ischemic lesion was observed on C) T2‐WI and D) T2*‐WI. Red arrowheads indicate hypointense signals in the ischemic lesion of the pMCAo mouse. E) Percentage of T2 hypointense signals inside the ischemic lesion of mice subjected to pMCAo but not injected with NanoGd (group II, *n* = 4 mice) on baseline and post‐NanoGd (D3) MRI. F) Percentage of T2 hypointense signals inside the ischemic lesion of mice subjected to pMCAo and injected with NanoGd (group I, *n* = 14 mice). On these boxplots, the “X” represents the mean and the circles show the interior or outlier points. Significant differences between experimental groups on paired Student's *t*‐test are indicated by *** for *p* < 0.001. Gd‐DOTA: Dotarem. w/o: without. Ns: nonsignificant.

During this longitudinal protocol, mice were imaged by MRI 24 h following stroke (baseline session), which was induced by permanent middle cerebral artery occlusion (pMCAo). Immediately after, they were injected with NanoGd and reimaged 48 h later (post‐NanoGd MRI session) (Figure 2A). On baseline T2‐WI, cortical edema was detected in all mice subjected to pMCAo surgery, indicative of the ischemic lesion (dotted white line, **Figure** **3**A). After Dotarem injection, contrast enhancement was systematically observed in the ischemic area of pMCAo mice on baseline images (white arrowheads, Figure 2B), indicative of BBB disruption. Two pMCAo mice could not be imaged with pre‐ and post‐Dotarem T1 sequences due to technical reasons. In sham‐operated animals, no cortical lesions or BBB leakage were seen (Figure 3A,B).

Two mice died before the post‐NanoGd MRI session (Figure 2B: group I, *n* = 1; group III, *n* = 1), due to experimental reasons related to mouse anesthesia during intravital microscopy sessions, and three mice were excluded from quantitative analyses due to the small size of the ischemic lesion at D1, which was not representative of the pMCAo model (group I, *n* = 2/16 mice I; group II, *n* = 1/5). Infarct size was stable between baseline and post‐NanoGd MRI: 15 [11; 17] mm^3^ versus 14 [11; 17] mm^3^ (*p* = 0.969) (*n* = 18, pooled from group I and group II). Of note, NanoGd did not alter infarct size at D3 (group I: 14 [11; 15] mm^3^ vs group II: 15 [14; 17] mm^3^, *p* = 0.581) (Figure S1, Supporting Information). T2‐WI and T2*‐WI acquired 48 h after NanoGd injection (D3 post‐pMCAo) showed strong hypointense MR signals within the ischemic lesions of the 16 pMCAo mice in group I (red arrowheads, Figure 3C,D). These hypointense signals were not found in pMCAo mice not injected with NanoGd (group II) or in sham‐operated mice (group III). For all animals in group I, hypointense MR signals were mainly detected in the ischemic core. Examples of this spatial distribution are shown in Figure S2 in the Supporting Information in four representative pMCAo mice. Quantification of these MR hypointense signals in the ischemic lesion showed that, in group I, they covered 44% [36%; 51%] of the ischemic lesion at D3 post‐pMCAo (Figure 3F), which was significantly more than in group II at D3 post‐pMCAo (8% [5–11%], *p* = 7.62E‐7, paired *t*‐tests; Figure 3E) and compared to group I baseline hypointense signals (21% [15–29%], *p* = 7.46E‐5; Figure 3F). In studies using USPIO‐enhanced MRI, the extent of T2 hypointense signals has been shown to positively correlate with the quantity of magnetic nanoparticles on the one hand and with the area of F4/80+ macrophages on the other hand.^[^ ^24^ ^]^ The presence of hypointense signal on baseline T2‐WI corresponds to artifacts resulting from slight extracranial bleeding associated with pMCAo surgery (Figure 3A; Figure S2A, Supporting Information, black arrowheads). In group I, visual analysis of MR images of the 14 pMCAo mice that received both Gd‐DOTA and NanoGd showed complete colocalization of T1 enhancement at baseline and T2 hypointense signals (post‐NanoGd MRI) in *n* = 6/14 (43%) mice (see example in Figure S2B,C in the Supporting Information, mouse #4), partial colocalization in *n* = 7/14 (50%) mice (see examples in Figure S2B,C in the Supporting Information, mice #2 and #3), and no colocalization in *n* = 1/14 (7%) mouse (Figure S2B,C, Supporting Information, mouse #1). Of note, extralesional hypointense MR signals were also detected in the corpus callosum and external capsule in 9/16 (56%) mice of group I (yellow arrows, Figure S2C,D, Supporting Information).

### 2.3 Two‐Photon Intravital Microscopy Demonstrates NanoGd Internalization by CX3CR1‐GFP/+ Cells

**Figure 4:**
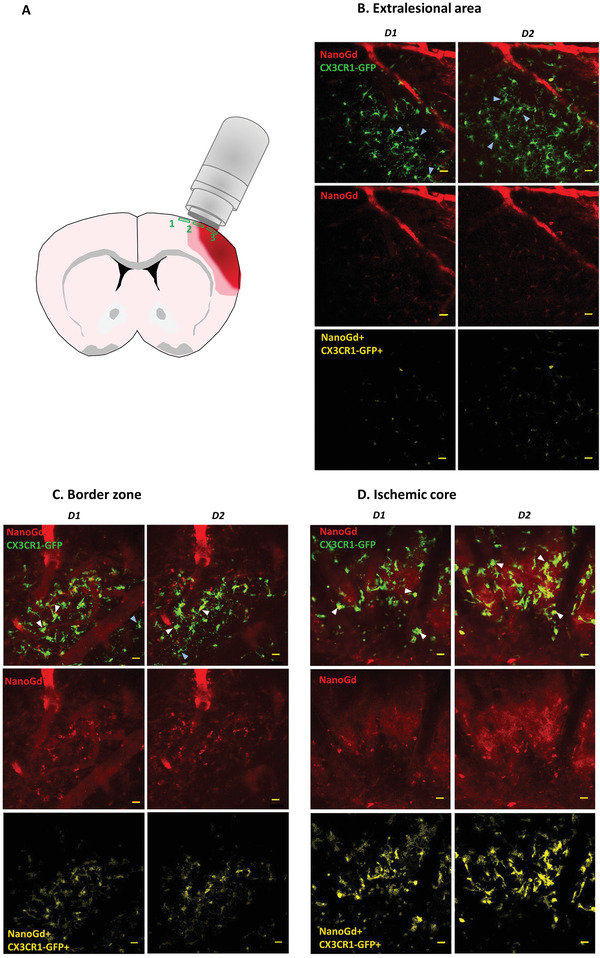
Visualization of NanoGd interaction with CX3CR1‐GFP/+ cells in the ischemic brain with intravital two‐photon microscopy. A) Schematic representation of the ischemic brain for a pMCAo mouse. The three areas imaged with two‐photon microscopy are represented (green boxes): 1 = extralesional area; 2 = border zone; 3 = ischemic core. B–D) Representative images of two‐photon microscopy for a pMCAo mouse injected with NanoGd, for the first (D1) and the second imaging session (D2). For each subregion, images on the top present fluorescence signals from CX3CR1‐GFP/+ cells (in green) and NanoGd (in red), images in the middle represent fluorescence signals from NanoGd only (in red), and images on the bottom show colocalization between CX3CR1‐GFP/+ cells and NanoGd signal (in yellow). Examples of highly ramified CX3CR1‐GFP/+ cells are indicated by light blue arrowheads, and white arrowheads indicate less‐ramified/large body CX3CR1‐GFP/+ cells. (Scale bar: 20 µm.)

To understand the biological substrates of MR hypointense signals, we examined NanoGd fate in the 6 mice of group I, in 2 sessions of intravital two‐photon microscopy (Figure 2). For each mouse, we imaged the same three cortical areas at D1 and D2 following pMCAo: 1) extralesional area, 2) border zone, and 3) ischemic core (**Figure** **4**A). Due to difficulty in positioning the cranial implant to encompass all three regions together, the extralesional area was imaged in *n* = 4/6 mice and the ischemic core in *n* = 5/6 mice. For each area, we selected regions with a large number of parenchymal CX3CR1‐GFP/+ cells, to better study their interactions with NanoGd; therefore, cell density does not accurately reflect global cell density associated with pMCAo in these 3 subregions. Moreover, we distinguished two types of CX3CR1‐GFP/+ cells: “circulating CX3CR1‐GFP/+ cells,” characterized by a small round‐shaped morphology and highly mobile profile, which we assume to be present in perfused cerebral vessels or perivascular spaces, and “parenchymal CX3CR1‐GFP/+ cells,” which seem to be located in the parenchyma, associated either with a large cell body or with a small round‐shaped morphology, and with a less dynamic profile.

We first investigated CX3CR1‐GFP/+ cell density and morphology independently of the presence of NanoGd. In group I, the density of CX3CR1‐GFP/+ circulating cells was higher in the ischemic core than in the border zone, and higher in the border zone than in the extralesional area, with a significant effect of the “brain area” factor (Figure S3A, Supporting Information). Circulating cell density did not vary between D1 and D2 in the extralesional area (Figures S3A and S4, Supporting Information). In contrast, the density of parenchymal CX3CR1‐GFP/+ cells was similar in all three sampled regions and did not vary between D1 and D2 (Figure S3B, Supporting Information), except in the ischemic core where it significantly increased (Figure S4, Supporting Information). In the three subregions, parenchymal CX3CR1‐GFP/+ cells presented different cellular morphologies, indicative of different activation status. The extralesional area was characterized by highly ramified parenchymal CX3CR1‐GFP/+ cells with small cell body (light blue arrowheads in Figure 4B and movies S1 and S2 in the Supporting Information), the ischemic core contained less‐ramified parenchymal CX3CR1‐GFP/+ cells with large cell bodies (white arrowheads in Figure 4D and movies S3 and S4 in the Supporting Information), and the border zone contained cells with both phenotypes (Figure 4C and movies S5 and S6 in the Supporting Information). These cell phenotypes were observed independently of NanoGd administration (Figure S5A–C, Supporting Information). Parenchymal CX3CR1‐GFP/+ cell morphology assessment confirmed the visual observations, showing a significant increase in cell body area, accompanied by a strong decrease in process area and ramification index (IR) from extralesional area to ischemic core both at D1 and D2 (Figure S3C–E, Supporting Information). Of note, these parameters were measured in 5 to 6 cells per mouse, and then compared between the 6 mice of group I imaged with two‐photon microscopy. We observed a significant mouse effect for these three morphological parameters (cellular body surface, *p* = 0.002; process surface, *p* = 3.826E‐15; ramification index, *p* = 6.421E‐05).

**Figure 5:**
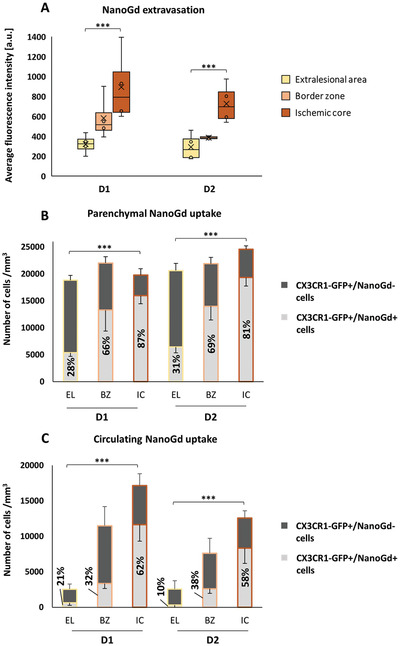
Two‐photon quantitative analyses reveal NanoGd brain diffusion and internalization by CX3CR1‐GFP/+ cells in the ischemic lesion. Quantification of NanoGd brain diffusion and cell internalization at D1 and D2 for pMCAo mice with complete two‐photon intravital imaging: extralesional area, border zone, and ischemic core (group I, *n* = 4). A) Graphic representation of NanoGd fluorescence intensity inside brain interstitium. The “X” represents the mean and the circles show the interior or outlier points. Number of B) parenchymal and C) circulating CX3CR1‐GFP/+ cells that internalized NanoGd (CX3CR1‐GFP+/NanoGd+ cells, light gray) or not (CX3CR1‐GFP+/NanoGd− cells, dark gray). Data are displayed as mean ± SD and the percentage of CX3CR1‐GFP/+ cells that internalized NanoGd is also indicated. For all graphs, significant differences between brain areas and imaging days were calculated on two‐way ANOVA and are indicated as *** for *p* < 0.001. There was no significant effect of imaging day (D1 vs D2). For the “NanoGd uptake” graphs, statistical analysis was performed on the CX3CR1‐GFP+/NanoGd+ cell percentage. a.u.: arbitrary unit.

Following NanoGd injection, visual analysis of two‐photon images showed NanoGd extravasation from blood sector to brain parenchyma. Interstitial red fluorescence decreased from the ischemic core, where it was clearly seen, to the border zone and extralesional area, where it was almost unnoticeable (NanoGd fluorescence images on Figure 4B–D and movies in the Supporting Information). Quantitative analysis of the interstitial red fluorescence was performed to confirm these observations. We measured the fluorescent signal in pMCAo mice not injected with NanoGd (group II) to evaluate the level of autofluorescence. Results showed that interstitial red signal was near zero in both the extralesional and ischemic areas in the absence of NanoGd. In contrast, a strong fluorescent signal was found in the ischemic core interstitium at D1 and D2 and, to a lesser extent, in the border zone in pMCAo mice injected with NanoGd (group I) (**Figure** **5**A). In the extralesional area, fluorescence intensity was above autofluorescence levels and there was significant enhancement of red fluorescence in the border zone and ischemic core (Figure 5A). In parallel, we observed and subsequently quantified NanoGd internalization by CX3CR1‐GFP/+ cells. A large proportion of parenchymal CX3CR1‐GFP/+ cells internalized NanoGd particles in the ischemic core, slightly fewer in the border zone, and very few in the extralesional area (yellow signal on the images on the right, Figure 4B–D). These observations were supported by quantitative analyses of the percentage of CX3CR1‐GFP+/NanoGd+ cells, with more than two‐thirds of parenchymal CX3CR1‐GFP/+ cells on average having internalized NanoGd in the whole ischemic lesion (border zone and core; Figure 5B). The percentage of parenchymal CX3CR1‐GFP+/NanoGd+ cells varied little between D1 and D2 in all three subregions (Figure 5B; Figure S4, Supporting Information). Circulating CX3CR1‐GFP+/NanoGd+ cells showed the same decreasing gradient from ischemic core to extralesional area, as observed in parenchymal CX3CR1‐GFP+/NanoGd+ cells, with half the circulating CX3CR1‐GFP/+ cells on average having internalized NanoGd in the whole ischemic lesion (border zone and ischemic core; Figure 5C). Intravital two‐photon imaging of sham‐operated mice injected with NanoGd (group III) showed no morphological evidence of CX3CR1‐GFP/+ cell activation, negligible NanoGd leakage in the brain parenchyma and very little NanoGd internalization by parenchymal CX3CR1‐GFP/+ cells (Figure S5B, Supporting Information).

Visual observation of CX3CR1‐GFP/+ cell interaction with NanoGd suggested several hypotheses about the two types of CX3CR1‐GFP/+ cells identified in our analyses in the ischemic zone: parenchymal CX3CR1‐GFP/+ cells that are larger and highly connected to each other, and likely correspond to activated microglia (white arrowheads in Figure 4C,D, and Figure S6 and movies in the Supporting Information); in contrast, we also identified CX3CR1‐GFP/+ cells that are small round‐shaped and nonramified. Highly mobile round‐shaped CX3CR1‐GFP/+ cells may correspond to monocyte‐derived macrophages recruited to the lesion site, whereas the less dynamic ones may correspond either to infiltrated monocyte‐derived macrophages present within the brain parenchyma or to activated microglia (white arrows in Figure S6 and movies in the Supporting Information). We also detected patches of red signal that seemed to correspond to NanoGd accumulation in CX3CR1‐GFP/− cells, in both the ischemic core and the border zone (blue arrows in Figure S6 and dark blue arrows in movies in the Supporting Information).

### 2.4 Postmortem Analyses Confirm NanoGd Presence in the Ischemic Lesion and Internalization by Phagocytic Cells

**Figure 6:**
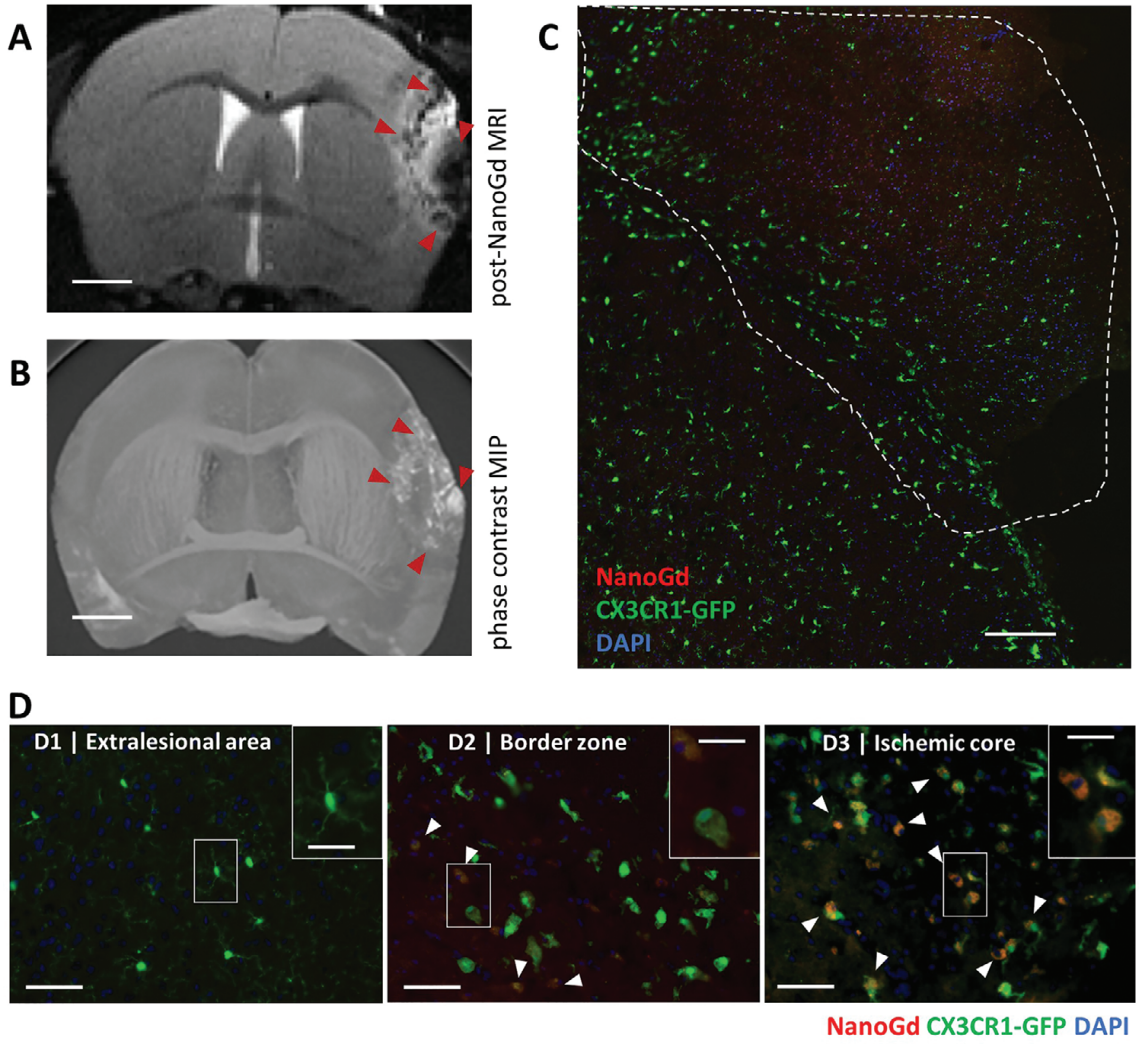
Spatial distribution of NanoGd following pMCAo is confirmed by ex vivo analysis. A) Hypointense signals on in vivo T2‐WI colocalized with hyperintense signals observed on B) maximum intensity projection (MIP) obtained from ex vivo X‐ray phase‐contrast image, used as gold standard to map Gd distribution. Red arrowheads indicate these signals in the ischemic lesion. Both images were obtained from the same pMCAo mouse injected with NanoGd (scale bar: 1 mm). C,D) Fluorescence microscopy images obtained from brain sections of a representative pMCAo mouse. C) The ischemic lesion is delineated by a dotted white line on a macroscopic view of the ischemic hemisphere (scale bar: 250 µm). D) Higher magnification shows CX3CR1‐GFP/+ cells and NanoGd in the D1) extralesional area, D2) border zone, and D3) ischemic core. White arrowheads indicate the area of colocalization between CX3CR1‐GFP/+ cells and NanoGd (scale bars: 50 µm for overview images; 20 µm for magnified insets).

To make sure that MR signal drops did not come from NanoGd within brain capillaries, mice were euthanized by intracardiac perfusion to remove any NanoGd particles that might remain in the blood sector. Using high‐resolution X‐ray phase‐contrast images, hyperintense signals corresponding to gadolinium (from NanoGd) distribution were visualized inside the ischemic core (red arrowheads, **Figure** **6**B). These signals colocalized with in vivo hypointense MR signals (red arrowheads, Figure 6A).

Histological analyses highlighted a spatial distribution of CX3CR1‐GFP/+ cells quite similar to that described on intravital microscopy: 1) small cell body/highly ramified CX3CR1‐GFP/+ cells characteristic of resting‐state microglia in extralesional areas and close to the border zone; 2) large cell body/less‐ramified CX3CR1‐GFP/+ cells surrounding the ischemic core, corresponding to the border zone; and 3) both large cell body/less‐ramified and small round‐shaped CX3CR1‐GFP/+ cells with no ramifications (likely circulating cells) in the ischemic core (Figure 6C,D). Separate observation of each area showed that in the ischemic core of pMCAo mice injected with NanoGd, very round‐shaped CX3CR1‐GFP/+ cells were often associated with red fluorescent signal, corresponding to NanoGd (white arrowheads, Figure 6D3). Higher magnification suggested an intracellular location for NanoGd (magnified inset, Figure 6D3). In the border zone, CX3CR1‐GFP/+ cells also presented a round‐shaped phenotype and some colocalized with NanoGd (white arrowheads and magnified inset, Figure 6D2) whereas in the extralesional area, CX3CR1‐GFP/+ cells were not associated with red fluorescence (Figure 6D1). In sham‐operated mice, histological staining showed resting‐state microglia in the cortical area but no NanoGd (Figure S5C, Supporting Information).

**Figure 7:**
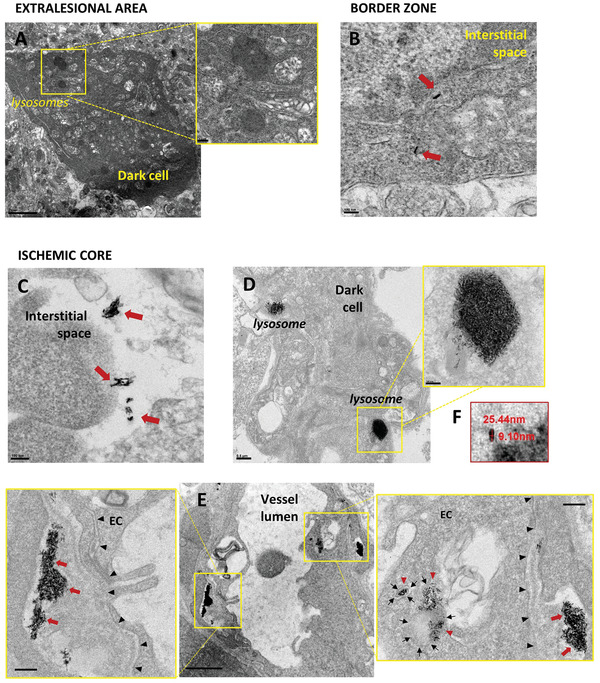
Characterization of NanoGd subcellular distribution using transmission electron microscopy. A–C) Transmission electron microscopy (TEM) images from three brain areas of a pMCAo mouse injected with NanoGd: extralesional area, border zone, and ischemic core. A) Focus on an immune phagocytic cell (dark cell) in the extralesional area. Higher magnification shows absence of electron‐dense structures inside the lysosomes (scale bar: 2 µm for overview image; 200 nm for magnified inset) B) Isolated electron‐dense structures detected within the interstitial tissue of the border zone, indicated by red arrows (scale bar: 100 nm). Images from the ischemic core show accumulation of single and aggregated electron‐dense structures C) inside the interstitial space (red arrows; scale bar: 100 nm), D) inside phagocyte lysosomes (scale bar: 0.5 µm for overview image; 100 nm for magnified inset), and E) inside the endothelial cells (EC) that surround cerebral vessels (scale bar: 1 µm). Higher magnification of the cerebral vessel wall (magnified insets in (E)) shows endothelial cells and their basal lamina membrane (black arrowheads), which delineates the extremity of the cerebral endothelium (scale bar: 200 nm). In these magnified insets, NanoGd is detected in endothelial cell cytoplasm (red arrowheads) inside cytoplasmic vesicles identified by the presence of biological membrane (black arrows), and outside endothelial cells as indicated by red arrowheads (panels (B) and (C)). F) Higher magnification of the ischemic core TEM image shows that the maximal height of the electron‐dense structure is around 25 nm.

Finally, TEM was performed on brain samples extracted from one representative pMCAo mouse injected with NanoGd (group I). In the border zone, single electron‐dense structures with the same size as the NanoGd were found within dark cells full of lysosomes, identified as microglia/macrophages, and within interstitial tissue (**Figure** **7**B, red arrows). In the sample from the ischemic core, electron‐dense structures were sometimes detected alone or in small aggregates in the interstitial space (Figure 7C). More importantly, electron‐dense structures were massively present inside the lysosomes of dark cells (Figure 7D) and in the endothelial cells that surround cerebral vessels (Figure 7E). To gain some insight into brain ingress mechanisms, special attention was paid to NanoGd distribution around the cerebral vessels in the ischemic core. An accumulation of electron‐dense structures was detected inside endothelial vesicles and in the brain interstitium on the other side of the basal lamina (magnified insets, Figure 7E), suggesting at least partial endothelial transcytosis of NanoGd. Analysis of ischemic core TEM images at higher magnification showed that these electron‐dense structures were rice‐grain‐shaped and matched NanoGd's hydrodynamic size (Figure 7F). Energy dispersive X‐ray spectrometry (EDX) analysis confirmed that gadolinium was present in the ischemic core sample (Figure S7A, Supporting Information). In contrast, electron‐dense structures were not found in TEM images of the extralesional sample (example in Figure 7A) and this result was confirmed by EDX analysis showing absence of gadolinium in this tissue (Figure S7B, Supporting Information).

## 3 Discussion

The present study developed and thoroughly validated a multimodal protocol to image phagocytic cells in vivo in a mouse model of permanent cerebral ischemia. Efficient internalization of NanoGd by phagocytic cells was first established in vitro using primary cultures of microglial cells, confirming results previously obtained on primary BMDM culture.^[^ ^22^ ^]^ In vivo MRI demonstrated systematic T2/T2* hypointense signals in the ischemic lesion of pMCAo mice. Intravital two‐photon microscopy performed back‐to‐back with MRI demonstrated in vivo internalization of NanoGd by CX3CR1‐GFP/+ cells. Postmortem TEM analysis confirmed that NanoGd accumulated within lysosomes of phagocytic cells. All these data, taken together with our results showing the absence of toxic effects of NanoGd on brain cells and tissue, confirm the potential of NanoGd‐enhanced MRI as an imaging biomarker of immune phagocytic cells at the subacute stage of ischemic stroke.

This study builds upon and extends previous studies of USPIO‐enhanced MRI for monitoring phagocytic cells in vivo in ischemic stroke, which showed hypointense signals in the ischemic lesion and in remote areas such as the corpus callosum and external capsule.^[^ ^24^, ^25^, ^26^, ^27^ ^]^ We used intravital two‐photon microscopy back‐to‐back with MRI to decipher the working mechanisms of nanoparticle‐induced MR signals. This has not previously been done to the best of our knowledge, although understanding what is imaged is instrumental for a wide spread application of the technique, beyond stroke studies and even beyond neurological applications.^[^ ^28^ ^]^ Intravital two‐photon microscopy is a powerful method, complementary to noninvasive 3D imaging techniques, providing the spatial and temporal resolution required to monitor the dynamics of immune cells in the central nervous system.^[^ ^10^, ^29^, ^30^ ^]^ Given the reproducible cortical localization of the pMCAo infarct, intravital microscopy is ideal to investigate immune cell involvement in this model, but few studies have used it in pMCAo mice.^[^ ^29^, ^31^ ^]^ To the best of our knowledge, our study is the first to provide longitudinal in vivo data using transgenic mice subjected to pMCAo and expressing enhanced green fluorescent protein (eGFP) under the control of fractalkine receptor (CX3CR1) promotor.

In CX3CR1eGFP mice, both microglia and monocyte‐derived macrophages express CX3CR1 and thus GFP,^[^ ^32^, ^33^ ^]^ therefore we were not able to distinguish them based on cellular fluorescence. Furthermore, the establishment of a classification based solely on morphological considerations seems imprecise: more and more publications highlight the possibility of a change in the phenotype of monocytes‐derived macrophages toward a microglial phenotype following cerebral injury.^[^ ^34^ ^]^ We therefore opted for an analysis based on the mobility of the cells, allowing us to distinguish between highly mobile cells and cells considered to be stationary. This classification has already been used by other teams for the analysis of different leukocyte populations present in the brain.^[^ ^31^, ^35^ ^]^ Visual observation of CX3CR1‐GFP/+ cell morphologies and mobilities highlighted the presence of two distinct populations: 1) small round‐shaped CX3CR1‐GFP/+ cells with a highly mobile profile, essentially in the ischemic core, which are most probably recruited and/or infiltrated monocyte‐derived macrophages,^[^ ^36^ ^]^ and 2) activated CX3CR1‐GFP/+ microglial cells with large cell body and few ramifications with a less dynamic profile, in the ischemic core and border zone.^[^ ^7^, ^29^ ^]^ These results are in line with previous research on pMCAo mice, showing in postmortem specimens the presence and activation of immune phagocytic cells at the lesion site from D1 to D5 following ischemic stroke.^[^ ^37^, ^38^, ^39^ ^]^ Using the same pMCAo model as in our study in LysM‐eGFP mice, Zarruk et al. showed that the level of infiltrated myeloid cells peaked at 72 h postischemia and that they were confined in the ischemic core surrounded by a dense region of microglia.^[^ ^39^ ^]^ In addition, they reported that the core of the lesion contained rounded microglia, and concluded that “microglia and macrophages are both involved in the phagocytosis of the damaged tissue in cortical ischemic lesion, a prerequisite for subsequent repair processes.”^[^ ^39^ ^]^ In agreement with these findings, we observed an increase in parenchymal CX3CR1‐GFP/+ cell density in the ischemic core from D1 to D2 and morphological changes toward a rounder/less‐ramified phenotype from the extralesional lesion to the ischemic core. Another interesting result of our intravital two‐photon microscopy study was the spatiotemporal changes in CX3CR1‐GFP/+ circulating cell density. There was a clear ascending gradient of CX3CR1‐GFP/+ circulating cells from the periphery to the core of the lesion, consistent with stronger recruitment of monocyte‐derived macrophages at the lesion core.^[^ ^39^ ^]^ A recent study in hyperacute stroke patients also showed increased leukocyte circulation at the proximity of the ischemic lesion compared to remote regions.^[^ ^40^ ^]^ Interestingly, blood samples were collected beyond the occlusion site and before clot removal. Retrograde collateral pial blood flow may explain the presence of monocytes downstream of the occlusion. In models of permanent ischemia, the question of how monocyte‐derived macrophages reach the unperfused ischemic core remains little explored.^[^ ^41^ ^]^ Our data, showing for the first time to our knowledge the presence of circulating CX3CR1‐GFP/+ cells in the ischemic heart, suggests that their entry route might be via retrograde collateral pial blood flow. Thus, independently of NanoGd applications, the current study provides invaluable insight into microglia/macrophage response collected in vivo and longitudinally at the subacute stage of ischemic stroke.

Administration of NanoGd led to reproducible hypointense signals that were systematically detected with in vivo MRI in the ischemic core of the lesion at 72 h postischemia: i.e., in regions with intense phagocytic activity at this stage, as described above.^[^ ^39^ ^]^ In the current study, we have explored this time point only, as it corresponds to a plateau for both lesion size and macrophage activation.^[^ ^19^, ^42^ ^]^ Because NanoGd‐enhanced MRI is minimally invasive, earlier and later time points may be investigated longitudinally in future work. NanoGd accumulation within ischemic lesions of pMCAo mice may be explained by two distinct mechanisms, which are probably complementary. The first has been described in the literature as the “Trojan horse mechanism,” by which NanoGd is internalized in the vascular sector by activated peripheral phagocytes secondly recruited at the ischemic lesion by inflammatory signals. The second consists in NanoGd crossing the BBB, followed by secondary internalization by immune phagocytes present at the lesion site (microglia and/or infiltrating macrophages).^[^ ^25^, ^43^ ^]^ Intravital two‐photon microscopy showed an interstitial red fluorescence signal in the ischemic core and, to a lesser extent, in the border zone, suggesting NanoGd extravasation in these regions. Small aggregates of nanoparticles were also detected on TEM in the interstitium of the ischemic core. In addition, two‐photon microscopy showed uptake of NanoGd by the two CX3CR1‐GFP/+ cell populations present: large cell body/less‐ramified microglia, and small round‐shaped monocytes/macrophages. One concern may be related to the presence of interstitial NanoGd in the ischemic core, which may induce confounding MR signals, related to BBB dysfunction rather than to uptake by immune phagocytic cells. However, previous studies showed that T2/T2*‐weighted sequences were more sensitive to nanoparticle‐laden macrophages than to free nanoparticles.^[^ ^19^, ^44^ ^]^ The current study further demonstrated in vivo that a vast majority of CX3CR1‐GFP/+ cells in the ischemic core (two‐thirds of circulating and four‐fifths of parenchymal CX3CR1‐GFP/+ cells) were loaded with NanoGd. In addition, highly mobile NanoGd‐laden CX3CR1‐GFP/− cells were also detected in the ischemic core. We hypothesized that these cells might be neutrophils, which are CX3CR1‐negative immune cells present at the lesion at the acute and subacute stages of permanent stroke, with the potential to internalize magnetic nanoparticles.^[^ ^38^, ^45^, ^46^ ^]^ Therefore, we assume that NanoGd‐laden phagocytic cells are the main contributors of MR signal changes and that hypointense regions seen on NanoGd‐enhanced MRI correspond to areas with high immune phagocytic activity. One way to tackle the issue of signal specificity would be to perform a longitudinal study at later time points, to monitor NanoGd wash‐out from the interstitial space and evaluate impact on MR hypointense signal changes over time.

Unexpectedly, some interstitial red fluorescence (above autofluorescence level) was found in the extralesional area, suggesting NanoGd extravasation in this region also. Around one‐third of CX3XR1‐GFP/+ cells had internalized NanoGd in this area, although the intracellular fluorescence intensity was much less pronounced than in the lesion. Because of the small brain surface covered by the cranial implant, the imaged extralesional area was relatively close to the lesion (1–2 mm from the ischemic core). It is probable that the extralesional area is not “healthy” tissue and cannot be considered in a similar manner to contralateral brain tissue. This raises the important question of NanoGd accretion in the brain. The BBB was systematically permeable to Gd‐DOTA in the core of the lesion at the time of NanoGd administration. Although the two contrast agents do not have the same size, there may have been some paracellular influx of NanoGd through dysfunctional or destroyed tight junctions.^[^ ^47^ ^]^ Another pathway could be endothelial transcytosis, which is increased at the subacute stage of ischemic stroke and known to be a pathway for nanomedicine delivery.^[^ ^48^, ^49^, ^50^ ^]^ Our TEM results suggest that at least some NanoGd particles may enter the brain via this mechanism. Elucidating the brain delivery mode of NanoGd, for instance, by using in vitro models of the BBB, would be a crucial next step to better understand the mechanisms of phagocytic cells labeling. Nevertheless, we did not see any MR hypointense signal in extralesional areas, suggesting that NanoGd‐enhanced MRI detects regions with high phagocytic activity rather specifically. Such imaging tool is crucial for studying neuroinflammation at the subacute stage of ischemic stroke, as there is currently no other minimally invasive imaging alternative at this early stage.

Interestingly, the spatial distribution of NanoGd‐associated hypointense signals (mainly in the ischemic core) differed from that obtained with USPIO‐enhanced MRI in the same stroke model (mainly in the border zone), despite broadly similar experimental chronologies.^[^ ^19^, ^24^, ^25^ ^]^ Depending on the microenvironment, microglia/macrophages acquire different polarizations (from anti‐inflammatory M1 to proinflammatory M2) in the border zone and ischemic core.^[^ ^37^ ^]^ Thus, USPIO and NanoGd may in fact label populations of microglia/macrophages with different polarizations. NanoGd particles are quite similar to USPIOs in their relaxometry properties and zeta potential but have a distinct type of coating, which is known to strongly influence uptake by phagocytic cells.^[^ ^15^, ^51^, ^52^ ^]^ Unfortunately, in the present study, in vitro analysis and sample preparation for postmortem analyses did not allow to assess the phenotype of microglia/macrophages that internalized NanoGd. Future studies should aim at confirming this intriguing and exciting possibility, using, for instance, flow cytometry‐based phagocytosis assay for in vitro evaluation and histological co‐staining of the CD68 lysosomal marker, CD11b, Ym1, and CD206 for postmortem brain samples analysis.^[^ ^42^ ^]^

The originality of our method is based on a new multimodal nanoprobe, NanoGd. The NanoGd nanoparticle was designed to have a relatively small hydrodynamic diameter, long vascular remanence, and negative zeta potential, all properties known to be associated with greater uptake by macrophages.^[^ ^15^, ^53^ ^]^ In terms of relaxivity, NanoGd appears to be a T2/T2* contrast agent despite the presence of gadolinium.^[^ ^22^, ^54^ ^]^ One drawback of this T2/T2* property, however, is that other signal sources than the nanoparticles (microhemorrhage, for instance) may induce hypointense signals, hence, the need for a precontrast scan. The rationale for using a gadolinium‐based rather than an iron‐based contrast agent was the perspective of developing molecular imaging with novel emerging CT technologies such as phase‐contrast imaging (as used in the present study) and spectral photon‐counting CT (SPCCT). The latter represents the next generation of clinical CT and displays the unique feature of allowing specific imaging of materials with *k*‐edge energies in the 30–120 keV range (thereby including gadolinium but not iron). Importantly, this innovative approach would eliminate the need for a prescan. Due to their long vascular remanence, GdF_3_‐based contrast media have already been successfully used for performing angiography with SPCCT.^[^ ^55^ ^]^ In addition, SPCCT was recently shown to be very promising for monitoring and quantifying gold‐labeled macrophages in rat brain.^[^ ^56^ ^]^ More data are needed to fully assess the safety profile of NanoGd, especially with regards to a potential brain accumulation. Provided that those data confirm the absence of long‐term toxicity, NanoGd‐enhanced SPCCT may become an alternative imaging approach for the cellular and molecular imaging of inflammatory responses.

## 4 Conclusion

The present study described and validated a multimodal imaging method to monitor phagocytic cells following stroke in vivo, based on the injection of a novel nanoprobe, NanoGd. This method appears to be a promising tool for studying the spatiotemporal response of microglia/macrophages associated with subacute ischemic lesion at multiple scales. NanoGd‐enhanced imaging may serve as a preclinical in vivo surrogate marker of neuroinflammation, thus enhancing understanding of the pathophysiology of stroke and, potentially, improving monitoring of immunomodulatory treatments. Importantly, NanoGd may also help studying other neurological disorders, and more generally, other inflammatory pathologies.

## 5 Experimental Section

### NanoGd Nanoparticle

NanoGd is composed of a 16 nm magnetic core of gadolinium fluoride (GdF_3_), coated with bifunctional bisphosphonate polyethylene glycol (PEG) and functionalized for the present study with a Lemke‐type fluorophore (LEM‐A) for fluorescence imaging.^[^ ^22^ ^]^ NanoGd fluorescence excitation and emission wavelengths are respectively 510 and 682 nm, and the two‐photon excitation wavelength is 980 nm. NanoGd has a rice‐grain shape with 28 ± 8 nm hydrodynamic diameter and 0.251 ± 0.003 polydispersity index (PDI = [SD/mean]^2^). Its *r*1 and *r*2 relaxivities (measured at 7T in saline at ambient temperature) are 1 and 20 mm ^–1^ s^–1^, respectively; thus, NanoGd is a T2/T2* contrast agent.^[^ ^22^ ^]^ The zeta potential of NanoGd is −42 ± 6 mV at physiological pH.

### Animal Experiments

All experimental procedures involving animals and their care were carried out in accordance with the European regulations for animal use (EEC Council Directive 2010/63/EU, OJ L 276, Oct. 20, 2010) and the study was approved by the local review board (“Comité d’éthique pour l'Expérimentation Animale Neurosciences Lyon:” CELYNE; CNREEA no. 42; APAFIS agreement no. #4688). Data were reported according to ARRIVE guidelines (Animal Research: Reporting of In Vivo Experiments). Animals were housed in groups of six per cage (except for the two‐photon microscopy experiments: one animal per cage, due to the cranial implant), in a temperature and humidity‐controlled environment (21 ± 3 °C), with 12:12 h light–dark cycle, and free access to standard chow and tap water. Animal experiments were performed in 8 week‐old (24 ± 2 g) C57Bl/6 male mice (Janvier, France), and 8–12 week‐old (23 ± 2 g) CX3CR1^eGFP/+^ transgenic male mice (originally donated by Serge Nataf in 2012 and bred at the INMG Institute, Claude Bernard University, Lyon).

### Microglial Cell Culture

Microglia were isolated from postnatal P0–P1 C57Bl/6 pups and prepared for primary cell culture. Briefly, brains were removed and rinsed in phosphate buffer saline with 0.6% glucose (PBS‐glucose). After removal of the meninges, hemispheres were mechanically dissociated in PBS‐glucose. Cells were collected and centrifuged, and then seeded in DMEM medium with 10% fetal calf serum (FCS), which allows glial cell growth but not neurons. They were cultured at 37 °C in humidified 5% CO_2_/95% air and the medium was changed at D1, D3, and D7. Microglial cells were mechanically detached from the dish by agitation and freezing for collection at confluence (after 15–20 days). At this step, other glial cells remained attached to the dish. Cells were then replated for 24 h in 12‐well plates containing glass coverslips coated with poly‐l‐ornithine, at a density of ≈80 000 cells per well. The following day, cultures were incubated with either 0 × 10^−3^ m(control condition), 0.5 × 10^−3^ m, or 1.5 × 10^−3^ m NanoGd for 24 h to characterize dose‐dependent NanoGd behavior in vitro.

### Immunocytochemistry

To evaluate NanoGd internalization, microglial cells were stained directly on the glass coverslips. Briefly, cells were fixed with 4% paraformaldehyde (PFA), rinsed with PBS, and incubated 1 h with 0.30% Triton X, 1% bovine serum albumin (BSA), and 5% goat serum (GS) in PBS to block nonspecific antibody labeling and permeabilize the cell membrane. Microglia were stained using a rabbit anti‐Iba‐1 antibody (1:500; 019‐19741, FUJIFILM Wako, Richmond, VA, USA) overnight at 4 °C. Cells were then rinsed in PBS and incubated 90 min with an antirabbit secondary antibody labeled with Alexa fluor 488 (1:1000; A21433, Invitrogen, Carlsbad, CA, USA). Finally, cells were rinsed three times in PBS, mounted on slides following Hoechst staining, and investigated for the presence of NanoGd and Iba‐1 positive cells. Images were acquired using a Leica TCS‐SP5X confocal microscope (Leica Microsystems, Wetzlar, Germany).

### Cytokine Immunoassays

To measure cytokine production, microglia culture supernatants were collected 24 h after NanoGd incubation. A positive control for the production of proinflammatory cytokines was added as a fourth experimental condition: microglial cells, not exposed to NanoGd, were activated with 100 ng mL^–1^ of LPS (*Escherichia coli* 0111:B4; Sigma‐Aldrich, Saint‐Louis, MO, USA) for 24 h. Then, IL‐6 and TNF‐*α* production was measured in the supernatant using mouse IL‐6 (ThermoFisher, KMC0061, Waltham, MA, USA) and TNF*α* (ThermoFisher, BMS607‐3, Waltham, MA, USA) ELISA kits according to the manufacturer's protocol.

### In Vivo Study Design

At day 0 (D0), 24 mice underwent permanent occlusion of the middle cerebral artery (pMCAo) to induce a reproducible cortical lesion. Baseline MRI was performed at day 1 (D1) post‐pMCAo to document the presence of the lesion and any BBB disruption. NanoGd was administered intravenously immediately after baseline MRI, at 2 mmol kg^–1^, to 17 of the 24 operated mice (group I). The other 7 mice that underwent surgery did not receive NanoGd and served as controls (group II). Three sham‐operated mice (without MCA occlusion) received NanoGd the same day and at the same dose as group I (group III). From the total set of animals, 12 mice (group I: *n* = 7; group II: *n* = 2; group III: *n* = 3) were prepared for longitudinal intravital two‐photon microscopy. They were imaged on the day of NanoGd injection (D1 post‐pMCAo, ≈8 h following injection) and followed up the next day (D2 post‐pMCAo, ≈28 h following injection). Except for four mice (group II: *n* = 2; group III: *n* = 2) that were imaged only with intravital microscopy, all the mice (*n* = 23) underwent postcontrast MRI at day 3 (D3) post‐pMCAo (48 h following injection). All animals were sacrificed by intracardiac perfusion at the end of the experiment and brains were sampled for histology, transmission electron microscopy, or X‐ray phase‐contrast tomography for 3D depiction of gadolinium brain distribution.^[^ ^57^ ^]^ Sample size was determined so as to obtain enough data for a proof‐of‐concept study.

### pMCAo Model

Focal cerebral ischemia was induced at D0 in mice anesthetized with isoflurane (induction: 3.5%; surgery: 2%; ISO‐VET, Piramal Healthcare, Morpeth, UK), by permanent occlusion of the distal middle cerebral artery (pMCAo) with iron chloride (FeCl_3_), as previously described by Karatas et al.^[^ ^58^ ^]^ Briefly, the right MCA was exposed by temporal craniectomy and occluded by a 10% FeCl_3_‐soaked filter‐paper strip on the dura mater, over the trunk of the distal MCA, for 10 min. To alleviate pain, subcutaneous (s.c.) 0.05 mg kg^–1^ buprenorphine injection was performed prior surgery, and at the end of the surgery, the wound was covered with lidocaine. During surgery, body temperature was monitored with a rectal probe and maintained at 37 °C using a feedback‐regulated heating pad. Animals were allowed to wake up completely from anesthesia under a heating lamp and were checked for behavioral abnormalities and weight loss twice daily until the end of the in vivo experiments.

### Thinned‐Skull Cortical Window Preparation

Thinned‐skull cortical window preparation was performed 1 day after pMCAo. For surgery, mice were exposed to isoflurane anesthesia (induction: 3–4%; surgery: 1.5–2%) and mounted in a stereotaxic apparatus (D. Kopf Instruments). At the beginning of surgery, buprenorphine was administered (0.05 mg kg^–1^ s.c.) for postsurgery pain relief. During surgery, mice were placed on a heating pad, and body temperature was maintained at 37 °C. After the skull was thoroughly cleaned and exposed, a 6 mm diameter custom‐made polyamide cranial implant was glued in place. This material has an advantage over the widely used metal‐containing implants of being compatible with MR imaging. The implant was centered ≈−0.5 anterior and +2.5 lateral from Bregma, corresponding to the periphery of the lesion, and adjusted according to the baseline T2‐weighted MRI images disclosing the size of the lesion. Thus, the 5 mm diameter area encircled both extralesional and ischemic tissue. The skull was carefully thinned over this area using a high‐speed drill. To avoid heat‐induced tissue injury, the bone was continuously cooled by repeated application of cold sterile saline. When the desired 20–30 µm bone thickness was reached, a cover glass was placed on top of a thin layer of cyanoacrylate glue over the thinned skull. At end of surgery, animals were allowed to wake up completely from anesthesia under a heating lamp and were then housed individually.

### MRI

MRI was performed on a 7T horizontal‐bore Bruker Avance II rodent imaging system (Bruker Biospin, Ettlingen, Germany). Anesthesia was induced with a mixture of air and 3.5% isoflurane and animals were placed in an MRI‐compatible mouse cradle. During acquisition, anesthesia was maintained with 2% isoflurane. The respiratory rhythm was carefully monitored by a pressure sensor linked to a monitoring system (ECG Trigger Unit HR V2.0, RAPID Biomedical, Rimpar, Germany), and body temperature was maintained by circulating heated water. A 50 mm inner diameter birdcage coil was used for transmission and a 15 mm diameter surface coil for reception.

For each sequence, 25 slices were acquired, from olfactory bulb to cerebellum, using field of view (FOV) 20 × 20 mm^2^, slice thickness 500 µm, and matrix size 256×256; these parameters yielded an in‐plane resolution of 78 µm. The in vivo MRI protocol comprised the following axial sequences: a T1‐weighted gradient‐echo (GRE) FLASH sequence pre‐ and postinjection of a small chelate of gadolinium (Dotarem, Guerbet, France, 0.2 mmol kg^−1^) intravenously, TE/TR 3.5/350 ms, bandwidth 101 kHz, number of averages 3, acquisition time 4 min; a spin‐echo T2‐weighted image (T2‐WI), TE/TR 43.8/5000 ms, bandwidth 40 kHz, number of averages 6, acquisition time 12 min; and a T2*‐weighted gradient echo FLASH sequence (T2*‐WI), TE/TR 6/750 ms, bandwidth 40 kHz, flip angle (FA) 20°, number of averages 8, acquisition time 19 min.

### Two‐Photon Intravital Microscopy

For each imaging session, mice were anesthetized with intraperitoneal injection of a mixture of ketamine (100 mg kg^–1^) and medetomidine (1 mg kg^–1^), and placed on a heating pad, with body temperature maintained at 37 °C. Imaging used a two‐photon microscope (Bruker Ultima) with Insight 3X laser (Spectra Physics) tuned to 980 nm for simultaneous excitation of both fluorescent proteins, eGFP and LEM‐A. A 20× water‐immersion lens (0.95 N.A. Olympus) was used. Green and red fluorescence was separated using a 560 nm dichroic mirror coupled to 525/50 nm and 650/40 emission filters.

Images were acquired at a depth of 50–150 µm at 3 sites: 1) ischemic core, corresponding to the lesional area with a majority of activated CX3CR1‐GFP/+ cells; 2) border zone, corresponding to the area around the lesion core, containing both activated and ramified CX3CR1‐GFP/+ cells; and 3) extralesional area, corresponding to the zone furthest from the lesion and containing a majority of ramified CX3CR1‐GFP/+ cells. These sites were determined and selected by visual observation of the morphological characteristics of microglial cells and their localization with regard to the lesion on two‐photon imaging. Twenty‐four to 45 consecutive Z‐stacks, with field size ≈420 × 420 µm, were acquired every minute with step size of 1 µm. A typical recording lasted ≈10–15 min (10–15 Z‐stacks). To compare progression in CX3CR1‐GFP/+ cell density and morphology and NanoGd internalization by CX3CR1‐GFP/+ cells, the same areas were imaged at D1 and D2. Anatomical landmarks such as blood vessels were used to locate the same areas between the two days of imaging.

### Data Analysis

MRI: The volume of hypointense MR signals on T2‐WI in the ischemic lesion was used as a biomarker of NanoGd accumulation following pMCAo.^[^ ^24^ ^]^ For each mouse, hypointense signal volume in the lesion was normalized by ischemic lesion volume. Analysis was performed manually by a single blinded operator using ImageJ software (National Institute of Mental Health, Bethesda, MD, USA; imagej.nih.gox/ij/).

Two‐photon microscopy: Image processing and analysis were performed using ImageJ and custom‐written MatLab software (ver. R2018b). Prior to analysis, the first Z‐stack from each acquisition was adjusted as well as possible between D1 and D2 in all three axes. For CX3CR1‐GFP/+ cell density count, the first Z‐stack from each acquisition, systematically containing 24 images, was used to generate a maximum intensity projection. After uniformly adjusting the images to reduce background noise, the number of CX3CR1‐GFP/+ parenchymal and circulating cells was quantified manually in each field of view. The total number of parenchymal and circulating CX3CR1‐GFP/+ cells was then divided by the imaging volume corresponding to the parenchyma and blood vessels, respectively, in order to generate a measure of cell density for each compartment. For nanoparticle internalization analysis, first, baseline fluorescence intensity was quantified in the red channel in control CX3CR1^eGFP/+^ pMCAo mice not injected with the nanoparticle (group II). This baseline fluorescence corresponded to the signal in the red channel resulting from autofluorescence and/or leakage of signal from the green fluorophore into the red detection channel. Thus, a fluorescence baseline value was obtained for each region (extralesional area, border zone, and ischemic core). This value was then subtracted from the red channel in pMCAo mice injected with the nanoparticle, to obtain the fluorescence signal specific to the nanoparticle as accurately as possible. To quantify the number of CX3CR1‐GFP/+ cells that had internalized the nanoparticle, the AND function of Image Calculator (ImageJ) was used on the first Z‐stacks from both channels, to obtain a Z‐stack containing the colocalized signal from both channels. An average Z‐projection was generated, and the number of cells that had internalized the nanoparticle was counted manually. This number was subtracted from the total number of CX3CR1‐GFP/+ cells in the field, providing a percentage value. For nanoparticle quantification in the brain interstitium, four 20 × 20 pixel ROIs were delineated on the average intensity projection in the red channel. Mean fluorescence intensity was measured for each ROI at D1 and D2 and averaged for each imaging session. For 2D time‐lapse video generation, images were first corrected for drift in the *x*, *y*, and *z* axes during acquisition, using a custom‐written MatLab program. The correction was accomplished by shift estimation from the cross‐correlation peak by fast Fourier transform (FFT) between the first stack of each acquisition (reference Z‐stack) and the following Z‐stacks. After realignment, images were uniformly adjusted for contrast and brightness to reduce background noise and enhance contrast. For parenchymal CX3CR1‐GFP/+ morphology analysis, cell body and process area were quantified using a custom‐written MatLab program, termed cell body recognition (CBR) method. This method is based on two‐step morphological image processing to extract the cell body from the processes: 1) application of a recursive erosion operator to eliminate all processes, and 2) application of a reverse dilatation operator to reconstruct the cell body according to cell morphology. This method enables cell body area to be computed, and the process area is obtained by subtracting the cell body from the total cell area. The IR is defined as the ratio of the cell's perimeter to its area, normalized to that of a circle of the same area, as described by Madry et al.^[^ ^59^ ^]^

(1) $$\mathrm{IR}=\frac{\mathrm{Perimeter}/\mathrm{Area}}{2\times {\left(\pi /\mathrm{Area}\right)}^{1/2}}$$

### Confocal microscopy

Confocal microscopy was used to investigate NanoGd internalization by cultured microglial cells in vitro. Five representative areas of 246 × 246 µm^2^ were acquired (6–9 Z‐stacks) for each slide for each condition (control, NanoGd 0.5 × 10^−3^ m, and NanoGd 1.5 × 10^−3^ m). NanoGd internalization by microglial cells in these areas was quantified on ImageJ using the method described above (Two‐photon microscopy in the “Data Analysis” section under the Experimental Section).

### Histology

Mice were euthanized by intracardiac perfusion with PBS followed by perfusion with 4% PFA. Brains were then removed, postfixed with 4% PFA for 24 h, and frozen in methylbutane with dry ice. Finally, tissues were cut into 12 µm sections by cryostat. For fluorescence analyses, slides were rinsed 3 times in 0.5% PBS‐Triton (PBST) and then mounted with Roti‐Mount Fluocare with DAPI. Then the slices were investigated for the presence of NanoGd and CX3CR1‐GFP/+ cells. Images were acquired from the 12 µm section using an Axio Scope A.1 fluorescence microscope (4 filters, Carl Zeiss, Oberkochen, Germany) equipped with a x0.63 AxioCam MRc (Carl Zeiss, Oberkochen, Germany).

### X‐Ray Phase‐Contrast Tomography

Ex vivo X‐ray phase contrast tomography was performed on brains prepared with the laboratory protocol.^[^ ^60^ ^]^ Briefly, mice were euthanized by intracardiac perfusion with PBS followed by 4% PFA. Brains were then extracted and dehydrated in successive ethanol baths: 25% (24 h), 50% (24 h), 75% (24 h), and 96% ethanol (24 h). Finally, mouse brains were placed inside 2 mL syringes to maintain them in a static position. In‐line phase contrast tomography was performed on beamline ID17 of the European Synchrotron Radiation Facility (ESRF) in Grenoble at 35 keV. An indirect detection‐based detector with a LuAg scintillator, standard microscope optics, and 2560 × 2160 pixel CMOS camera was positioned 11 m from the sample to obtain phase contrast. The whole‐brain data set was acquired at 6.1 µm isotropic pixel size. The acquisition time of the 2800 projections lasted <5 min per brain. Reconstruction was performed with Paganin's algorithm by setting *δ*/*β* to 1000, following Marinescu et al.^[^ ^57^ ^]^

### Transmission Electronic Microscopy

Mice were euthanized by intracardiac perfusion with PBS and then with a specific fixation solution (4% PFA, 4% glutaraldehyde, 0.3 mol L^−1^ cacodylate, 0.3 mol L^−1^ CaCl_2_). Brains were removed, and three samples of 1 mm^3^ were collected and stored in the fixation solution: one in the extralesional area, one in the border zone, and one in the ischemic core, each from the ipsilateral hemisphere. Finally, sample preparation for TEM imaging session was performed by the CIQLE platform (Medicine Faculty, Lyon, France) as described in the Experimental Section in the Supporting Information. Sections were examined with a Jeol 1400JEM (Tokyo, Japan) 120 kV transmission electron microscope equipped with a Gatan Orius 600 camera on wide‐field position and Digital Micrograph software (Gatan Inc.). EDX analysis was used to determine the presence of gadolinium within the TEM samples.

### Statistical Analysis

Data were not preprocessed and were presented either as box plots (median (25% percentile; 75% percentile)) or as histograms (mean ± standard deviation (SD)). One‐way, two‐way, and three‐way analyses of variance (ANOVA; R statistical software version 3.3.3 (2017‐03‐06) “Another Canoe”) were performed to evaluate the effects of: i) experimental condition; or ii) imaging day, imaged brain area, and (as appropriate) imaged mice. Statistical testing was done at two‐tailed alpha level of 0.05. A Student's *t*‐test (BiostaTGV website, Institut Pierre Louis UMR 1136 and UPMC, France) was performed to assess significant differences between: i) experimental groups, or ii) D1 and D2. Sample size for each statistical analysis was indicated in the figure legends. In all comparisons, a *p*‐value ≤ 0.05 was considered statistically significant.

## Conflict of Interest

This project is part of a public–private partnership with a firm that may market the investigated nanoparticle (MATHYM). Otherwise, the authors declare that the research was conducted in the absence of any commercial or financial relationships that could be construed as a potential conflict of interest.

## Supporting information

Supporting Information

Supplemental Movie 1

Supplemental Movie 2

Supplemental Movie 3

Supplemental Movie 4

Supplemental Movie 5

Supplemental Movie 6

## Data Availability

The processed data required to reproduce these findings and perform the statistical analyses are available to download at the figshare repository—https://figshare.com (https://doi.org/10.6084/m9.figshare.13046291). The raw data required to reproduce these findings cannot be shared at this time due to the large size of confocal, MRI, and two‐photon microscopy data, but can be available from the corresponding author on reasonable request.
