# Supplementary material for: Multimodal Imaging with NanoGd Reveals Spatiotemporal Features of Neuroinflammation after Experimental Stroke
Source: Adv Sci (Weinh). 2021 Jul 1;8(17):2101433. doi: 10.1002/advs.202101433 (PMC8425862; doi:10.1002/advs.202101433)
Supplement: Supplementary file 1 — Supporting Information [file ADVS-8-2101433-s002.pdf]

© 2021 Wiley-VCH GmbH

ADVANCED SCIENCE
Open Access

Supporting Information

for *Adv. Sci.,* DOI: 10.1002/advs.202101433

Multimodal imaging with NanoGd reveals spatiotemporal features of neuroinflammation after experimental stroke

*Violaine Hubert, Ines Hristovska, SzilviaKarpati, Sarah Benkeder, Arindam K Dey,Chloé Dumot, Camille Amaz, NauraChounlamountri, Chantal Watrin, Jean-Christophe Comte, Fabien Chauveau, Emmanuel Brun, Patrice N Marche,FrédericLerouge, Stéphane Parola, Yves Berthezène, Thomas Vorup-Jensen, Olivier Pascual, and Marlène Wiart**

Supporting Information

Multimodal imaging with NanoGd reveals spatiotemporal features of neuroinflammation after experimental stroke

*Violaine Hubert, Ines Hristovska, Szilvia Karpati, Sarah Benkeder, Arindam K Dey, Chloé Dumot, Camille Amaz, Naura Chounlamountri, Chantal Watrin, Jean-Christophe Comte, Fabien Chauveau, Emmanuel Brun, Patrice N Marche, Fréderic Lerouge, Stéphane Parola, Yves Berthezène, Thomas Vorup-Jensen, Olivier Pascual, and Marlène Wiart****

Experimental Section

Preparation of transmission electronic microscopy samples

All pieces of tissues were rinsed 3 times in 0.2 M sodium cacodylate buffer at 4°C. Samples were post-fixed with 1% aqueous osmium tetroxide for 1 hour, dehydrated in graded series of ethanol, and transferred to propylene oxide. Impregnation was performed with Epon epoxy resin (Leica EMPT automate, Leica Microsystems, Wetzlar, Germany). Inclusion was obtained by polymerization at 60°C for 72 h. Ultrathin sections (approximately 100 nm thick) were cut on a Leica UC7 ultramicrotome (Leica Microsystems, Wetzlar, Germany), mounted on 200 mesh copper grids, stabilized for 1 day at room temperature and contrasted with uranyl acetate.

Supporting Figures

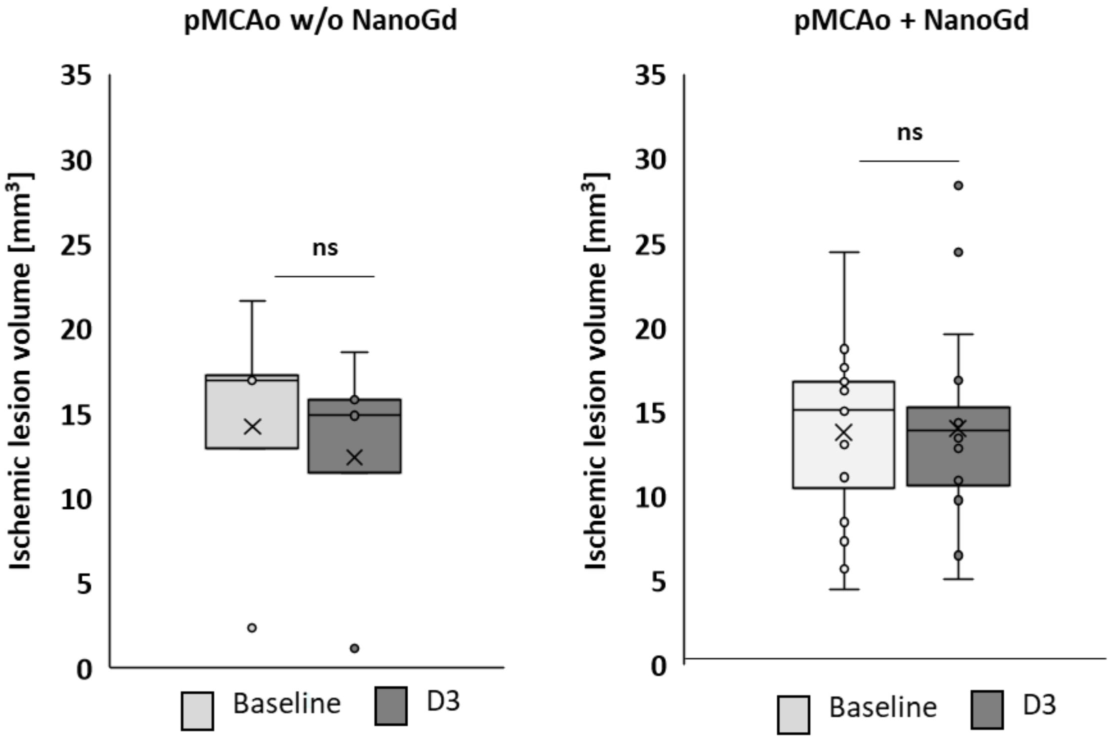

Figure S1. NanoGd injection does not increase the ischemic lesion size. Quantification of changes in ischemic lesion volume on MRI T2-WI acquired at 24 hours (baseline) and 72 hours (D3) post-pMCAo is similar for pMCAo mice not injected with NanoGd (group II, n=4) and for pMCAo mice injected with NanoGd (group I, n=14). The “X” represents the mean and the circles show the interior or outlier points. Significant differences between baseline and D3 were evaluated on paired Student t-test. ns: non-significant.

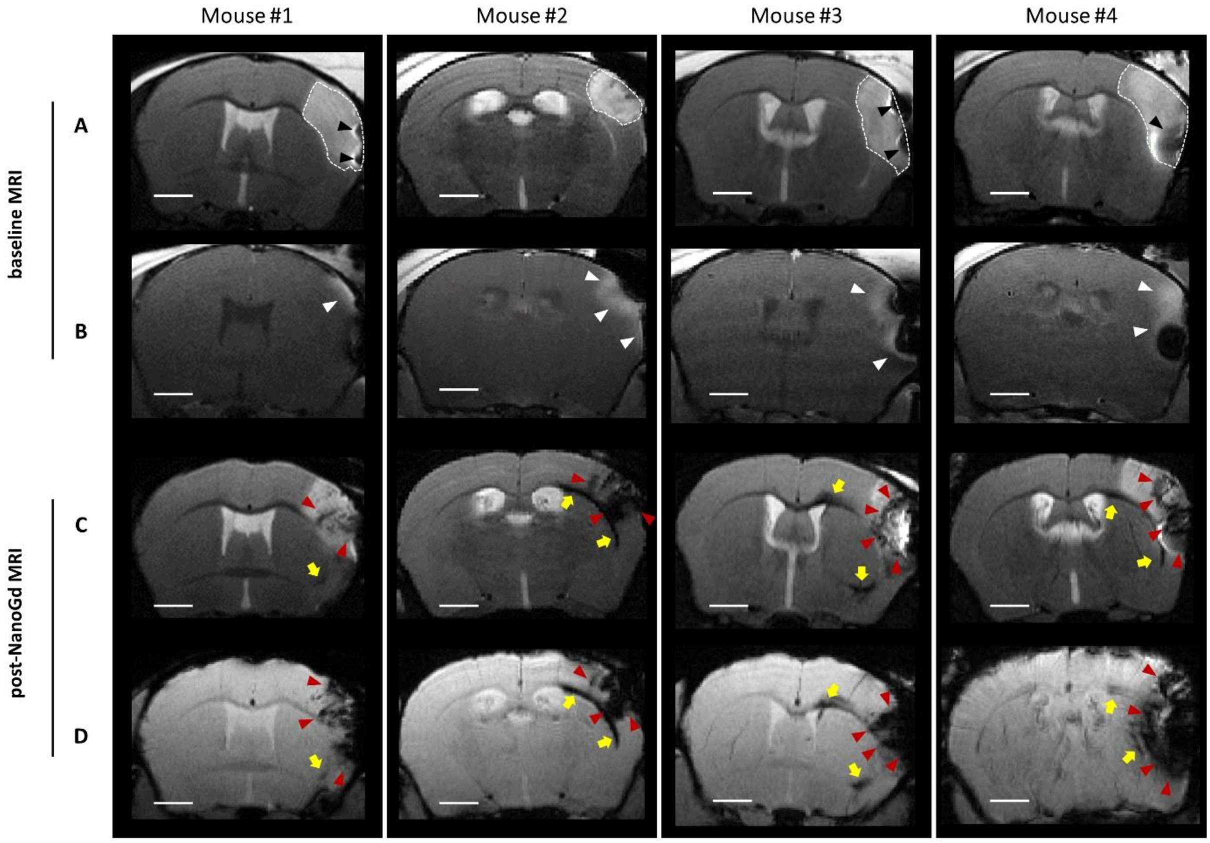

Figure S2. Heterogeneity of NanoGd distribution pattern following pMCAo. A-D. Baseline MRI (A-B) and post-NanoGd MRI (B-D) for 4 representative pMCAo mice injected with NanoGd. For each MRI sequence, only one transversal slice is shown. **A.** Dotted white lines delineate the ischemic lesion on baseline T2-WI, and black arrowheads indicate dark MR artefacts associated with pMCAo surgery. **B.** White arrowheads indicate T1 enhancement following Gd-DOTA injection on T1-weighted images, characteristic of BBB disruption. **C.** On post-NanoGd T2-WI, red arrowheads indicate the hypointense signals within the lesion, indicative of NanoGd presence. Yellow arrows indicate hypointense signals remote from the lesion, along the corpus callosum and external capsule. **D.** The same legend is used for post-NanoGd T2*-WI. (Scale bars: 1mm).

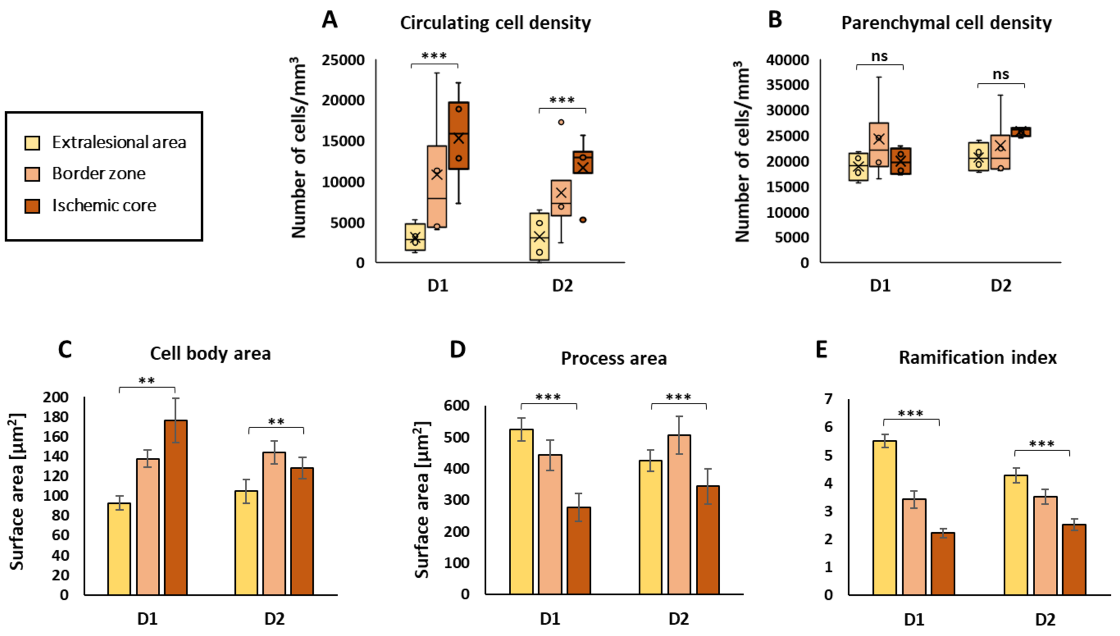

Figure S3. Characterization of CX3CR1-GFP/+ cell density and morphology following pMCAo with intravital two-photon microscopy. A-B. Quantification of circulating CX3CR1-GFP/+ cell density (A) and parenchymal CX3CR1-GFP/+ cell density (B) in the extralesional area, border zone and ischemic core at D1 and D2 post-pMCAo for pMCAo mice with complete two-photon intravital imaging: i.e., imaged at D1 and D2 in all 3 regions (group I, n=4). On these boxplots, the "X" represents the mean and the circles show the interior or outlier points. Significant differences between brain areas and imaging days were calculated on 2-way ANOVA and are indicated by *** for p <0.001. No significant effect of the imaging day (D1 versus D2) was found. **C-E.** Quantification of cell-body surface area (C), process surface area (D) and ramification index (E) for CX3CR1-GFP/+ cells in the extralesional area, border zone and ischemic core at D1 and D2 post-pMCAo. For each pMCAo mouse imaged with intravital microscopy (group I, n=6), 5 to 6 cells per brain area were analyzed. Data are displayed as mean ± SD. Significant differences between brain areas and the imaging days were calculated on 3-way ANOVA and are indicated by ** for p <0.01 and *** for p <0.001. No significant difference was found between imaging days (D1 versus D2). ns: non-significant.

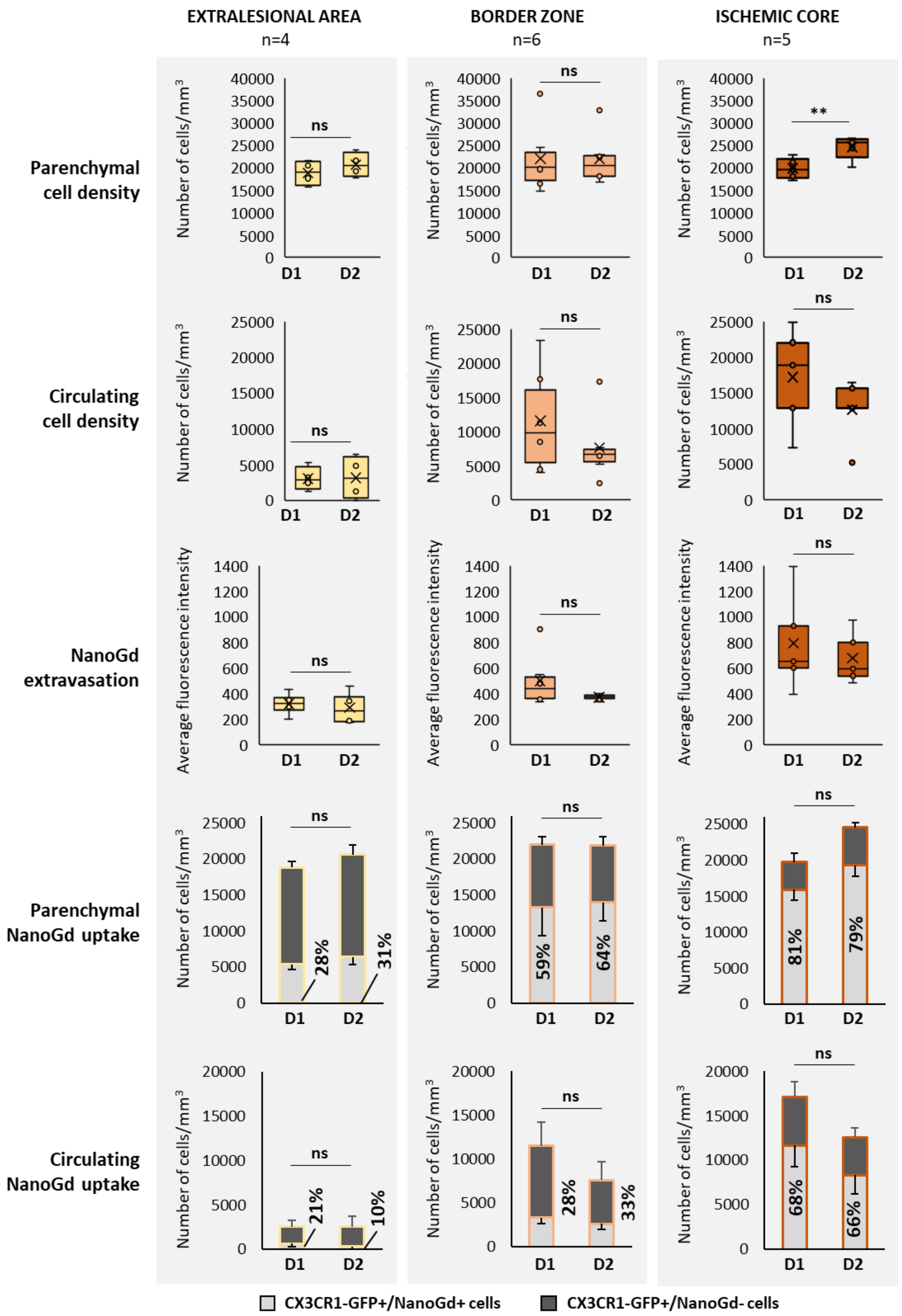
EXTRALESIONAL AREA
n=4
BORDER ZONE
n=6
ISCHEMIC CORE
n=5
Parenchymal cell density
Circulating cell density
NanoGd extravasation
Parenchymal NanoGd uptake
Circulating NanoGd uptake
Number of cells/mm^3
Average fluorescence intensity
D1
D2
ns
**
28%
31%
59%
64%
81%
79%
21%
10%
28%
33%
68%
66%
CX3CR1-GFP+/NanoGd+ cells
CX3CR1-GFP+/NanoGd- cells

Figure S4. Spatiotemporal evolution of CX3CR1-GFP/+ cells and NanoGd investigated with intravital two-photon microscopy. Comparison of CX3CR1-GFP/+ cellular density, NanoGd extravasation inside the brain interstitium and NanoGd internalization by CX3CR1-GFP/+ cells at D1 versus D2 post-NanoGd injection, in the extralesional area (EL), border zone (BZ) and ischemic core (IC) of pMCAo mice (EL: group I, n=4; BZ: group I, n=6; IC: group I, n=5). On these boxplots, the "X" represents the mean and the circles show the interior or outlier points. For the "NanoGd uptake" graphs, CX3CR1-GFP/+ cells that internalized NanoGd (CX3CR1-GFP+/NanoGd+ cells) are represented in light gray and CX3CR1-GFP/+ cells that did not internalize NanoGd (CX3CR1-GFP+/NanoGd- cells) in dark gray. The indicated percentage is the proportion of CX3CR1-GFP/+ cells that internalized NanoGd. For all graphs, significant differences were calculated on paired Student t-test and are indicated by ** for $p < 0.01$. ns: non-significant.

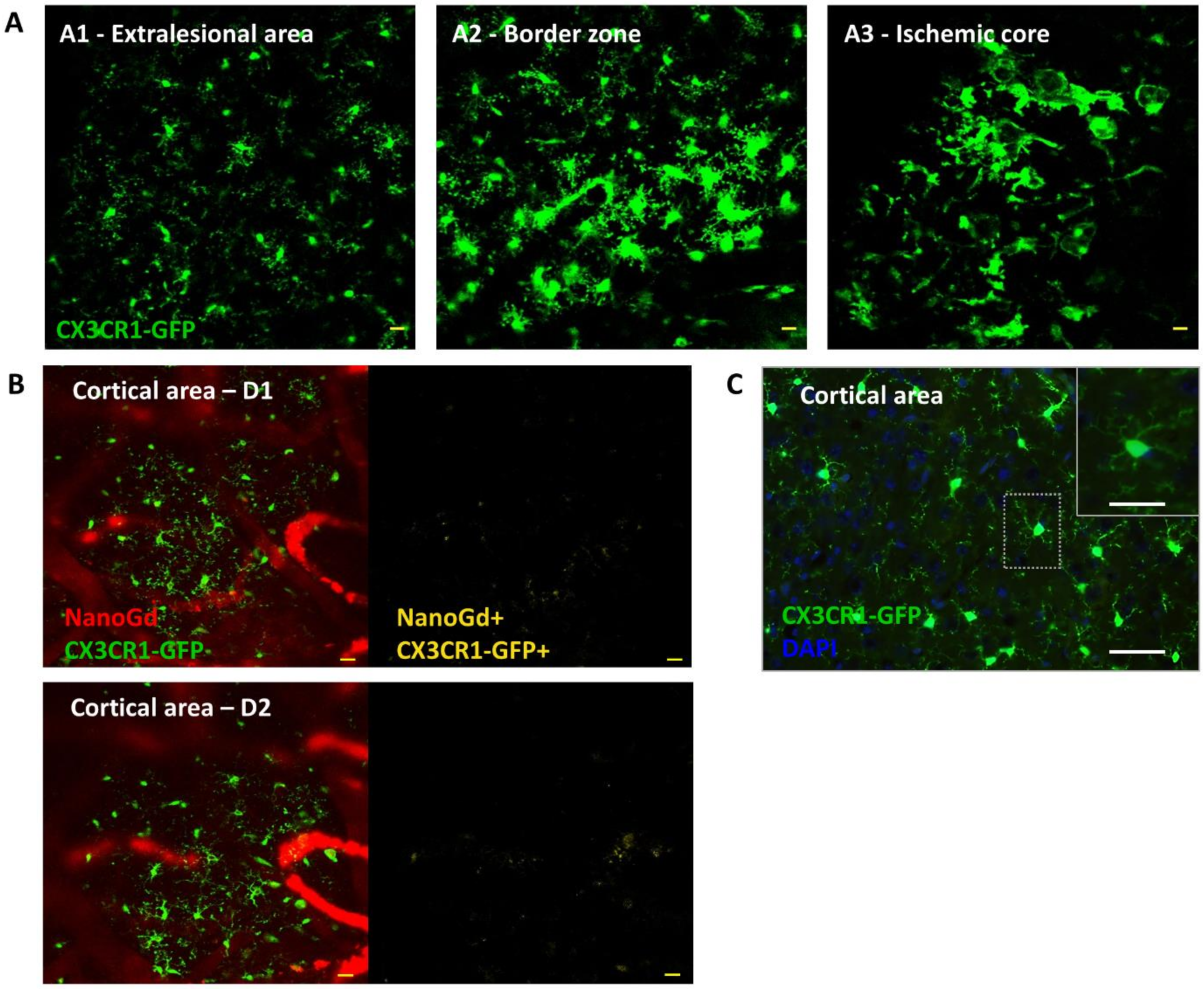

Figure S5. Characterization of NanoGd and CX3CR1-GFP/+ cell interaction in control groups with two-photon and fluorescence imaging. A. Representative images of two-photon microscopy for a pMCAo-operated mouse not injected with NanoGd, in the extralesional area (A1), border zone (A2) and ischemic core (A3) (scale bar: 20µm). **B.** Two-photon microscopy representative images of a cortical area located in the middle cerebral artery (MCA) vascular territory at D1 (upper panel) and D2 (lower panel) in a sham-operated mouse injected with NanoGd. Images on the right show two-photon signals from CX3CR1-GFP/+ cells (green) and NanoGd (red), and images on the left show areas where CX3CR1-GFP/+ cells and NanoGd signal colocalize (in yellow) (scale bar: 20µm). **C.** Fluorescence microscopy image of a sham-operated mouse injected with NanoGd shows ramified CX3CR1-GFP/+ cells in a cortical area located in the MCA vascular territory. NanoGd was not detected on these histological brain sections (scale bars: 50 µm for overview images; 10 µm for magnified insets).

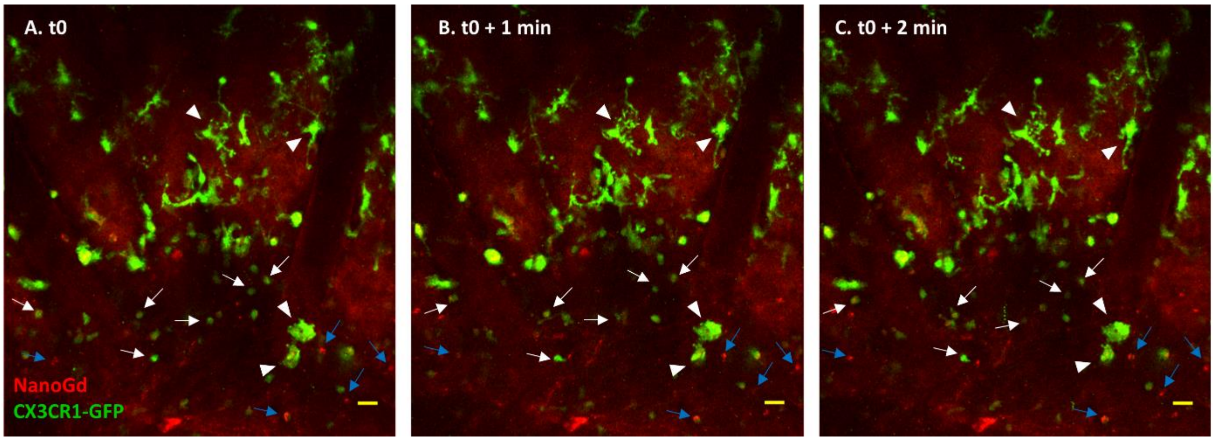

Figure S6. CX3CR1-GFP positive and negative cell populations within the ischemic core. A-C. Two-photon microscopy images from a time-lapse recording in the ischemic core of a representative pMCAo mouse. White arrowheads show several parenchymal CX3CR1-GFP/+ cells, assumed to be activated microglia cells. White arrows indicate a few small round-shaped CX3CR1-GFP/+ cells, which are likely to be recruited macrophages. Blue arrows indicate localized red signals that seem to correspond to NanoGd internalization in CX3CR1-GFP/- cells, which might be neutrophils. Time-lapse imaging shows that these cells are highly motile and/or mobile over time (scale bar: 20µm).

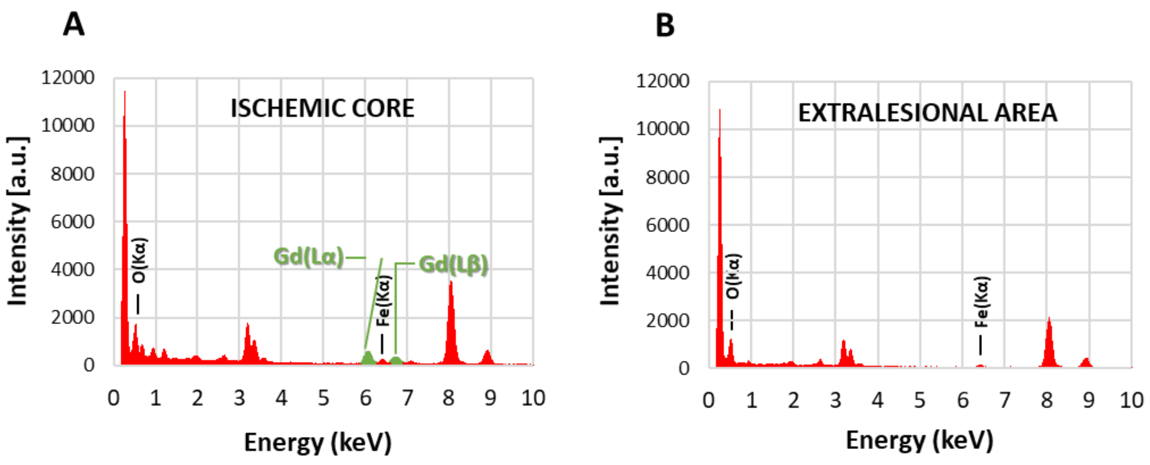

Figure S7. EDX analysis reveals gadolinium in the ischemic core following pMCAo. X-ray spectrometry analysis of extralesional area and ischemic core chemical composition, performed on TEM samples from a pMCAo mouse injected with NanoGd. **A.** In the ischemic core, 2 peaks characteristic of gadolinium (Gd) were detected at 6.053 keV (Gd Lα spectral line) and 6.708 keV (Gd Lβ spectral line). **B.** In contrast, no characteristic Gd peak was detected in the extralesional area. Constitutive chemical elements such as Oxygen (O) and Iron (Fe) were detected in both samples. Kα, Lα and Lβ: spectral lines characteristic of chemical elements. a.u.: arbitrary unit.

Supplementary online movies. **Two-photon time-lapse in vivo imaging of a representative extralesional area, border zone and ischemic core in pMCAo mice injected with NanoGd (group I)**. Movies of the extralesional area and ischemic core acquired at day 1 (D1) and day 2 (D2) comprise a 10-minute recording (10 Z-stacks), while movies of the border zone at D1 and D2 comprise a 14-minute recording (14 Z-stacks). Parenchymal CX3CR1-GFP/+ cells with large round-shaped morphology are indicated by white arrowheads, whereas ramified CX3CR1-GFP/+ cells assumed to be non-activated microglia are indicated by light blue arrowheads. Small round-shaped CX3CR1-GFP/+ cells which are likely to be recruited macrophages are indicated by white arrows. Finally, dark blue arrows indicate CX3CR1-GFP/- cells which could be neutrophils. (Scale bar: 20µm).
